# Psychological resilience as a selective perceptual amplifier in post-crisis learning: a dual-pathway model of academic motivation, perceived school support, and cognitive engagement – a multi-country study based on PISA 2022

**DOI:** 10.1186/s40359-026-04665-5

**Published:** 2026-05-01

**Authors:** Yuan Xiong, Mingjun Wang

**Affiliations:** 1 School of International Studies, Zhejiang Business College, Hangzhou, Zhejiang 310053 China; 2 College of Artificial Intelligence, Lishui University, Lishui, Zhejiang 323000 China

**Keywords:** Cognitive engagement strategies, Academic motivation, Perceived school support, Psychological resilience, Crisis education, PISA 2022

## Abstract

**Background:**

The global educational crisis triggered by the COVID-19 pandemic has posed persistent challenges to students’ deep learning. Understanding the mechanisms through which academic motivation (AM) and perceived school support (PSS) translate into cognitive engagement strategies (CES), and identifying factors that amplify this translation, is essential for building resilient educational systems.

**Methods:**

Data were drawn from the Programme for International Student Assessment (PISA) 2022, comprising 574,514 students from 80 countries/economies. Full Information Maximum Likelihood (FIML) estimation addressed missing data while retaining the full sample. Confirmatory factor analysis validated the measurement model. Design-corrected regression analyses incorporating PISA’s complex sampling weights and 80 replicate weights tested main effects and moderation hypotheses, controlling for ESCS. Mediation was tested using structural equation modeling with bootstrap confidence intervals.

**Results:**

AM significantly predicted CES both directly ($\beta$ = 0.089, $p<$ .001) and indirectly through enhanced PSS ($\beta$ = 0.221, $p<$ .001), with perceived support strongly predicting engagement ($\beta$ = 0.464, $p<$ .001). The indirect effect accounted for 53.6% of the total effect (bootstrap 95% CI [0.102, 0.104]). Psychological resilience (PR) significantly moderated the motivation$\rightarrow$support path ($\beta$ = 0.008, *p* = 0.007) but not the motivation$\rightarrow$engagement or support$\rightarrow$engagement paths. OECD membership attenuated both motivation$\rightarrow$support and motivation$\rightarrow$engagement paths, while gender only moderated the motivation$\rightarrow$engagement path.

**Conclusions:**

PR functions as a selective “perceptual amplifier” in post-crisis learning, primarily strengthening motivated students’ ability to recognize PSS. The robust support$\rightarrow$engagement pathway across national and gender groups underscores the universal importance of supportive school environments. Educational interventions could shift toward resilience-sensitive differentiated support.

## Introduction

Over recent years, global education systems have undergone a severe stress test. Large-scale school closures, the abrupt transition to online learning, and compounded family pressures have collectively created an unprecedented “crisis learning context.” This crisis has exposed not only disruptions in instructional delivery but also profound challenges to students’ intrinsic learning systems—including motivation, perceived support, and the capacity for deep cognitive engagement strategies. The PISA 2022 results indicate a significant decline in mathematical literacy across many countries, with average losses equivalent to nearly half a school year of formal instruction [1]. This trend reflects not only a setback in academic achievement but also the vulnerability of education systems to systemic shocks.

The profound impact of the pandemic on education extended beyond academic achievement, profoundly affecting the mental health and well-being of students and educators. School leadership has been identified as playing a critical role in fostering students’ stress resistance and emotional control during crises, highlighting the importance of the school-level environment [2]. However, while such macro-level factors are crucial, the ultimate efficacy of educational inputs hinges on students’ individual psychological resources and their internal learning processes. In the face of the systemic adversity of a global pandemic, a crucial question emerges: what psychological mechanisms enable some students to maintain deep cognitive engagement strategies despite disruptions, while others struggle?

Therefore, the core task of educational development urgently needs to shift from superficial “instructional recovery” to the deeper construction of “learning resilience” across all educational stages. Yet, what are the key mechanisms for building such resilience? Existing research primarily explains the sources of learning engagement from two perspectives: one emphasizing the central role of academic motivation (AM) as an internal driver [3], and the other highlighting the function of perceived school support (PSS) as an external scaffold [4]. However, within the psychological and contextual disturbances induced by crisis, the relationship between motivation and support may be more dynamic and intertwined. Specifically, motivation may prompt students to more actively perceive and utilize support resources. These positively perceived supports may, in turn, serve as “psychological fuel” for cognitive engagement strategies (CES). This potential “motivation $\rightarrow$ perceived support $\rightarrow$ cognitive engagement” pathway has not been sufficiently examined within crisis learning contexts.

This gap leads to the introduction of this study’s core moderating variable: psychological resilience (PR). Resilience entails not merely “bouncing back,” but represents a dynamic developmental system that enables positive adaptation through interactive processes across multiple levels [5]. Within the individual, it functions as a psychological regulatory system that influences how one interprets stress, mobilizes resources, and adapts to challenges. In educational settings, highly resilient students may function not only as “psychological shock absorbers” but also as “resource amplifiers”: they may be more adept at translating PSS into learning advantages. Recent research has shown that PR shapes adolescents’ sports participation by influencing exercise motivation, with variations across groups [6]. Building on this analogy, this prompts the question: In post-crisis academic learning, does PR similarly play a moderating role, enhancing the efficiency of transformation from motivation to perceived support and then to CES?

Situating the proposed moderation logic within a broader literature on how adversity-related experiences and psychological interpretations shape downstream outcomes provides further theoretical grounding. For instance, research in post-conflict settings demonstrates that traumatic war-related memories influence reconciliation partly through meta-dehumanization and dehumanization processes [7], and studies on ethnic identity show that responses to inclusion and exclusion depend on identity and belonging-related motivations [8]. These studies illustrate that contextual conditions often operate through subjective interpretation and personal psychological resources. Drawing on such work helps justify why resilience might condition the translation of motivation and perceived support into engagement, while also avoiding the impression that this argument emerges in isolation from neighboring theoretical traditions.

**Fig. 1:**
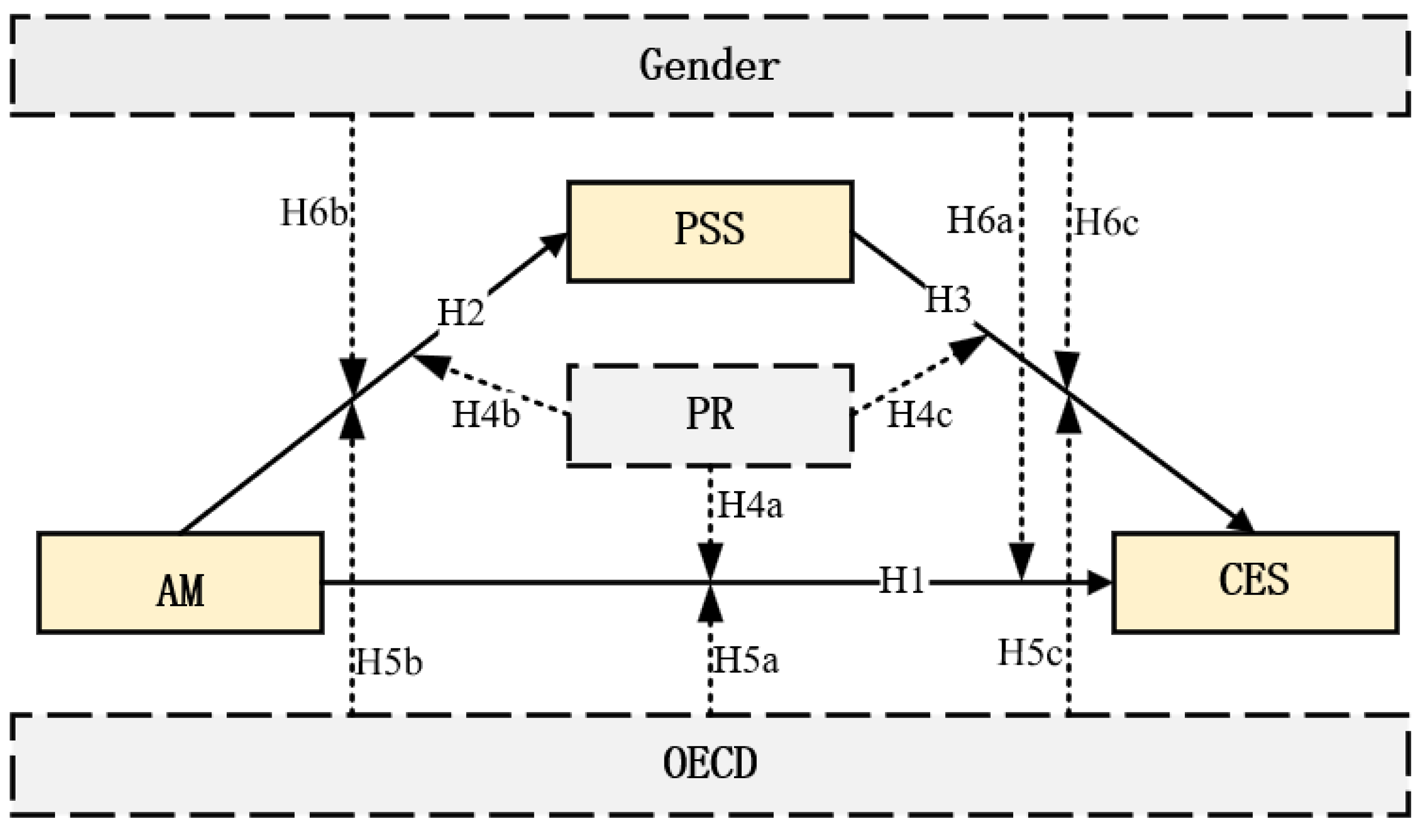
Conceptual model of the dual-pathway mechanism with tested moderators. Note*.* The model depicts hypothesized direct effects of AM on CES and PSS, and of PSS on CES. Dotted arrows represent hypothesized moderating effects of PR, OECD membership, and gender on the three core pathways

To address the aforementioned gaps, the present study constructs and tests a dual-pathway model with PR as a moderator, using multi-country PISA 2022 data. The conceptual model is presented in Fig. 1. The study aims to investigate:

- Does AM promote CES not only directly but also indirectly by enhancing PSS?
- Does PR significantly moderate the three paths: “AM $\rightarrow$ CES,” “AM $\rightarrow$ PSS,” and “PSS $\rightarrow$ CES”?
- Do macro-contextual variables (national development level proxied by OECD membership, and gender) moderate the core pathways?

The questions raised herein concern not only theoretical advancements in understanding educational resilience mechanisms but also practical orientations for post-crisis educational intervention: Should support continue in a “blanket” universal manner, or should it shift toward “targeted” resilience-sensitive designs? By systematically testing these questions on a large-scale multi-country sample, this study aims to provide empirically grounded insights for constructing more resilient, equitable, and effective educational systems.

## Literature review and theoretical framework

### CES in post-crisis educational development

As global education transitions from crisis response to long-term recovery, measures of progress must extend beyond surface indicators such as “return-to-school rates” or “compensatory instruction hours.” A scholarly consensus is emerging that the depth and quality of students’ CES is a key metric for assessing whether an education system has genuinely built learning resilience [4, 9]. CES is not passive reception of information; rather, it refers to a psychological process through which learners actively and strategically employ deep processing strategies (e.g., connecting, elaborating), metacognitive skills (planning, monitoring, regulating), and critical thinking to understand complex concepts and construct personal meaning [9]. In subjects like mathematics, this manifests as behaviors such as linking abstract concepts to real-world situations, flexibly applying reasoning to novel problems, and clearly explaining solution processes, which are hallmark features of deep cognitive engagement strategies [4]. The post-crisis learning environment remains fraught with uncertainty, and instructional continuity is often disrupted. In this context, students who can engage actively, deeply, and strategically are not only more likely to effectively navigate disruptions and mitigate learning losses but also internalize the core competencies required for lifelong learning. Therefore, investigating how to effectively stimulate and sustain CES in diverse post-crisis contexts constitutes a fundamental issue for educational research.

### Constructing a dual-pathway theory: linking internal drive and external support

How do students achieve deep cognitive engagement strategies? Existing theories primarily offer explanations from two parallel perspectives: one based on the internal drive of AM rooted in Self-Determination Theory, and the other emphasizing external support provided by schools as learning scaffolds. However, these perspectives often treat motivation and support as independent influences, overlooking the potential dynamic sequential relationship they may form in crisis learning contexts. Therefore, this study constructs an integrative “dual-pathway” theoretical model to more accurately reveal the complex mechanisms within post-crisis learning ecologies.

- **Pathway 1: The Direct Driving Effect of AM on CES**
  Rooted in Self-Determination Theory, AM—particularly motivation stemming from intrinsic interest and value identification—is considered the core engine that initiates and sustains deep learning strategies [3]. When students genuinely “want to learn well,” they are more inclined to adopt deep cognitive strategies that require greater mental effort. Meta-analytic evidence indicates that the correlation between intrinsic motivation and deep learning strategies is significantly higher than its correlation with surface strategies [10]. In the remote or hybrid learning modalities induced by crisis, this self-driven quality is particularly crucial, as it can motivate students to actively seek cognitive challenges even in the absence of direct supervision [11].
- **Pathway 2: The Influence of AM on PSS**
  Students’ motivational states act as cognitive filters, influencing how they interpret and evaluate their learning environment. Students with stronger learning motivation tend to view resources and assistance provided by the school more positively and openly, perceiving them as “scaffolds” conducive to learning rather than external burdens. Social Cognitive Theory emphasizes triadic reciprocal interactions among person, behavior, and environment, wherein individuals’ cognitive and belief systems (e.g., motivation) moderate how they attend to, interpret, and utilize environmental factors [12]. Thus, AM may not only directly drive learning behavior but also indirectly influence the learning process by shaping students’ subjective perception of PSS—that is, their evaluation of the help provided by the school in terms of autonomy, competence, and relatedness [3]. This subjective filtering role is particularly critical in the later stages of a crisis, when learning resources may be constrained and teacher-student communication may be less fluid than before, making students’ subjective perceptions often more influential than the objective availability of support.
- **Pathway 3: The Promoting Effect of PSS on CES**
  A substantial body of empirical research supports this pathway. Students’ perception of school support encompasses clear instructional structure and timely feedback (competence support), appropriate learning choices (autonomy support), and a caring classroom climate (relatedness support). When these needs are met, their psychological safety and academic self-efficacy are enhanced. This provides the necessary socio-psychological foundation for them to undertake complex learning activities that demand high cognitive load [4]. This is consistent with the meta-analytic finding that environmental support and positive teacher behaviors serve as key external promoting factors for student engagement [13]. Research during the pandemic indicates that structured online classes and asynchronous learning modules, if effectively meeting these basic psychological needs, can significantly promote students’ deep learning participation [14]. This cross-role evidence underscores the fundamental function of perceived support in fostering positive educational outcomes.

### PR as a key moderator: from “protective shield” to “amplifier”

The dual-pathway model outlined above depicts an ideal transmission mechanism from motivation and perceived support to CES; however, the strength of its effects likely varies across individuals. PR—defined as the dynamic psychological process through which individuals maintain positive adaptation or recover function swiftly in the face of adversity [5]—may be a core moderator determining the efficacy of this mechanism. Prior research has often treated resilience as a static “protective shield” that directly mitigates the negative effects of stress or predicts positive outcomes. This study reconceptualizes it as a “cognitive-affective amplifier” that may moderate the strength of the dual pathways by enhancing resource conversion efficiency. Highly resilient individuals typically possess better emotion regulation abilities and cognitive flexibility, which are key psychological assets that facilitate adaptive coping and positive reframing in the face of adversity [5]. When facing academic challenges, they can manage anxiety more effectively, directing attention and cognitive resources toward the task itself rather than expending them on threatening emotions.

Furthermore, broaden-and-build theory suggests that positive emotions—closely linked to resilience—broaden thought-action repertoires and build enduring personal resources [15]. Resilient students may experience more positive academic emotions even amidst crisis, which in turn facilitates deeper cognitive processing and more effective resource mobilization. This theoretical lens supports the hypothesis that PR amplifies the translation of motivation and support into engagement.

### Other potential moderators: developmental context and gender

To comprehensively understand the boundary conditions of the dual-pathway model, this study also includes two important contextual variables as moderators for exploratory analysis. National development level (proxied by OECD membership) serves as a macro-level proxy for the resource level and developmental stage of an education system. It is often hypothesized that in OECD countries, which typically have more abundant resources and more mature systems, the quality and accessibility of PSS are higher, and thus the support pathway may exert stronger effects. However, students in non-OECD countries may develop stronger PR or differentiated resource utilization strategies through coping with difficulties. Whether different developmental contexts lead to systematic differences in model pathways is an empirical question to be tested. Gender is also included as a potential moderator, as socio-cultural factors shape motivation orientations, stress-coping styles, and willingness to seek support resources among students of different genders.

### Research hypotheses

Based on the theoretical synthesis above, we propose an integrative conceptual model (see Fig. 1) to illustrate the dual-pathway mechanism of post-crisis learning recovery, with PR as a core moderator. The model posits that AM directly promotes CES and also enhances PSS, which in turn fosters CES. Crucially, PR is hypothesized to positively moderate the strength of all three direct pathways, acting as a personal resource amplifier. OECD membership and gender are also examined as exploratory moderators. The conceptual model with all tested paths is depicted in Fig. 1, which serves as the foundation for testing the following hypotheses:

Main Effect Hypotheses:

- H1: AM has a significant positive effect on CES.
- H2: AM has a significant positive effect on PSS.
- H3: PSS has a significant positive effect on CES.

Moderating Effect Hypotheses (PR):

- H4a: PR significantly and positively moderates the AM $\rightarrow$ CES path.
- H4b: PR significantly and positively moderates the AM $\rightarrow$ PSS path.
- H4c: PR significantly and positively moderates the PSS $\rightarrow$ CES path.

Moderating Effect Hypotheses (OECD Membership):

- H5a: OECD membership significantly moderates the AM $\rightarrow$ CES path.
- H5b: OECD membership significantly moderates the AM $\rightarrow$ PSS path.
- H5c: OECD membership significantly moderates the PSS $\rightarrow$ CES path.

Moderating Effect Hypotheses (Gender):

- H6a: Gender significantly moderates the AM $\rightarrow$ CES path.
- H6b: Gender significantly moderates the AM $\rightarrow$ PSS path.
- H6c: Gender significantly moderates the PSS $\rightarrow$ CES path.

## Methodology

### Data source and sample

This study utilizes data from the 2022 cycle of PISA, administered by OECD. The PISA 2022 assessment was conducted during the later stages of the global COVID-19 pandemic, and its survey framework specifically included modules designed to examine education systems’ crisis responses and students’ related experiences, making it a uniquely pertinent data source for investigating post-crisis learning recovery mechanisms [1, 16].

The initial student sample comprised 613,744 fifteen-year-old students from 80 participating countries and economies. To address missing data while preserving the full representativeness of the sample, we employed FIML estimation for all structural equation modeling analyses. FIML uses all available information from each case without listwise deletion, providing unbiased parameter estimates under the missing at random (MAR) assumption. The final analytical sample retained for model estimation comprised 574,514 students after accounting for missing data patterns, fully representing the PISA 2022 target population. Table 1 presents the demographic and descriptive characteristics of the analytical sample, including the distribution by OECD membership, gender, and the weighted means and standard deviations of the core study variables.

**Table 1:** Demographic and descriptive characteristics of the analytical sample

Characteristic	Value
Analytical sample size (N)	574,514
Original sample size	613,744
Number of countries/economies	80
OECD countries (%)	48.1%
Female (%)	50.4%
Age (years), mean (SD)	15.85 (0.29)
ESCS index, mean (SD)	-0.69 (1.24)
AM, mean (SD)	3.29 (0.71)
PSS, mean (SD)	2.67 (0.94)
CES, mean (SD)	3.00 (1.25)
PR, mean (SD)	3.28 (1.05)

Analytical sample size reflects the number of observations retained after FIML estimation ($N = 574{,}514$). Original sample size indicates all students in the PISA 2022 database with available sampling weights ($N = 613{,}744$). ESCS = Index of Economic, Social and Cultural Status. AM, PSS, CES, and PR are measured on 5-point scales. Weighted means and standard deviations are reported for scale scores where applicable

### Measures

The four core latent constructs involved in this study’s model all utilize established scales derived from the PISA 2022 assessment framework. These scales were constructed based on published PISA 2022 documentation and have demonstrated satisfactory reliability and validity in the cross-national crisis learning context [17].

- **AM**
  We measured AM using three items based on Self-Determination Theory, assessing students’ intrinsic goal orientation of “wanting to learn well” in mathematics, the test language, and science [3]. Students responded on a five-point Likert scale ranging from 1 (strongly disagree) to 5 (strongly agree). Example item: “I want to do well in my mathematics test.”
- **PSS**
  PSS directly addresses school responses during pandemic-related school closures. It was measured via three items asking about the frequency of school behaviors in following up on assignments, providing live online classes, and offering recorded learning materials. A five-point frequency scale was used. Example item: “How often did your school follow up on your assignments during school closures?”
- **CES**
  CES focuses on the mathematics learning context, measured via three items assessing whether teachers guide students to make real-world connections, apply logical reasoning, and explain solution processes. These items reflect instructional interactions that promote the use of deep cognitive and metacognitive strategies [9]. A five-point Likert scale was used.
- **PR**
  As the key moderating variable in this study, resilience is conceptualized as the capacity for positive adaptation and regulation in adversity [5]. It was measured via three PISA 2022 questionnaire items assessing emotional control, adversity recovery, and stress coping [16]. A five-point Likert scale was used.
- **Covariates and Grouping Variables**
  Students’ index of ESCS was included as a control covariate in all structural models. OECD membership (OECD vs. non-OECD) and gender (male vs. female) were used as grouping variables for multi-group moderation analyses.

### Analytical strategy

All data analyses were conducted using R version 4.5.3, following a rigorous multi-step procedure.

- **Measurement Model Evaluation**
  First, we assessed the construct validity of the four core latent variables via confirmatory factor analysis (CFA) using the lavaan package [18]. Standardized factor loadings, composite reliability (CR), and average variance extracted (AVE) were examined. Following recommendations by Hu and Bentler [19], model fit was evaluated using the scaled chi-square to degrees of freedom ratio ($\chi ^2/df$), Comparative Fit Index (CFI), Tucker-Lewis Index (TLI), Root Mean Square Error of Approximation (RMSEA), and Standardized Root Mean Square Residual (SRMR). Cronbach’s $\alpha$ coefficients were calculated on the full analytical sample to assess internal consistency reliability.
- **Main Effect and Moderation Testing with Complex Survey Design**
  To account for PISA’s complex two-stage stratified sampling design, all main effect and moderation analyses were conducted using design-corrected linear regression via the survey package in R [20]. Specifically, we incorporated student final sampling weights (W_FSTUWT), school-level clustering (CNTSCHID), and variance estimation via Fay’s Balanced Repeated Replication (BRR) with 80 replicate weights (W_FSTURWT1 to W_FSTURWT80), as recommended by the OECD PISA Data Analysis Manual [21].
  Main effects (H1–H3) were tested by regressing CES on AM and PSS, and PSS on AM. Moderation effects of PR (H4a–H4c) were tested by creating product terms between factor scores of AM and PR, and PSS and PR, and including these interaction terms in the regression models. Factor scores were obtained from the validated CFA model to circumvent convergence issues associated with full latent interaction modeling given the substantial missing data patterns. Moderation effects of OECD membership (H5a–H5c) and gender (H6a–H6c) were tested by including interaction terms between the respective grouping variable and the focal predictors in separate design-corrected regression models.
- **Mediation Analysis**
  The indirect effect of AM on CES through PSS was tested using structural equation modeling without survey design correction (due to bootstrap incompatibility with complex survey designs in current software implementations). Bootstrap confidence intervals based on 1,000 resamples (with 969 successful convergences) were obtained using the percentile method to assess the significance of the indirect effect.
- **Model Fit Criteria**
  Criteria for judging model fit were as follows: CFI $> 0.90$, TLI $> 0.90$, RMSEA $< 0.08$, and SRMR $< 0.08$ indicate acceptable to good model fit [19, 22].

## Results

### Measurement model assessment

Prior to testing the structural relationships, we assessed the reliability and validity of the measurement model. Results from confirmatory factor analysis (see Table 2) show that standardized factor loadings of all observed variables on their corresponding latent variables ranged from 0.429 to 0.852, all statistically significant at $p < .001$. These results meet the psychometric acceptable standard of factor loadings greater than 0.40, indicating that each measurement indicator effectively reflects its intended construct [23].

**Table 2:** Reliability and validity of measurement model

Construct	Item	Unstd.	S.E.	Z	p	Std.	$\boldsymbol{\alpha }$	CR	AVE
AM	AM1	1.000	–	–	–	0.847	0.872	0.873	0.696
	AM2	0.936	0.002	519.91	<.001	0.826			
	AM3	0.987	0.002	556.55	<.001	0.829			
PSS	PSS1	1.000	–	–	–	0.722	0.613	0.628	0.370
	PSS2	0.894	0.005	187.24	<.001	0.635			
	PSS3	0.595	0.004	144.16	<.001	0.429			
CES	CES1	1.000	–	–	–	0.736	0.706	0.736	0.495
	CES2	1.145	0.004	308.74	<.001	0.852			
	CES3	0.631	0.003	184.93	<.001	0.467			
PR	PR1	1.000	–	–	–	0.539	0.696	0.706	0.450
	PR2	1.319	0.007	185.85	<.001	0.662			
	PR3	1.620	0.008	199.58	<.001	0.788			

$N = 574{,}514$ after FIML. *Unstd*. unstandardized factor loading, *S.E*. standard error, *Std*. standardized factor loading, $\alpha$ Cronbach’s alpha calculated on the full analytical sample, *CR* composite reliability, *AVE* average variance extracted. All factor loadings are significant at $p < .001$

Regarding internal consistency reliability, Cronbach’s $\alpha$ coefficients for the latent variables ranged from 0.613 to 0.872, and composite reliability (CR) values ranged from 0.628 to 0.873. For convergent validity, the average variance extracted (AVE) for AM was 0.696, exceeding the 0.50 threshold, while AVE values for PSS (0.370), CES (0.495), and PR (0.450) were slightly below the conventional cutoff but considered acceptable in the context of large-scale international assessments with heterogeneous item content [24].

The overall measurement model demonstrated excellent fit to the data: $\chi ^2(48) = 11{,}413.28$, $p < .001$; CFI $= 0.986$; TLI $= 0.981$; RMSEA $= 0.020$ (90% CI [0.020, 0.021]); SRMR $= 0.031$. These fit indices collectively indicate a high degree of congruence between the theoretical measurement structure and the observed data across the 80 participating countries and economies (see Table 3).

**Table 3:** Model fit indices

Fit index	Value	Recommended Cutoff
$\chi ^2$ (Scaled)	11,413.28	–
*df*	48	–
$\chi ^2/df$	237.78	< 3.00
CFI	0.986	> 0.90
TLI	0.981	> 0.90
RMSEA [90% CI]	0.020 [0.020, 0.021]	< 0.08
SRMR	0.031	< 0.08

$N = 574{,}514$ after FIML. Fit indices are from the robust MLR estimator

### Main effect testing

The design-corrected regression analyses provided support for the direct pathways outlined in our conceptual model (see Table 4). AM had a significant positive effect on CES ($\beta = 0.089$, SE $= 0.003$, $t = 32.95$, $p < .001$), supporting H1. AM also had a significant positive effect on PSS ($\beta = 0.221$, SE $= 0.003$, $t = 87.55$, $p < .001$), supporting H2. PSS similarly significantly and positively predicted CES ($\beta = 0.464$, SE $= 0.004$, $t = 112.65$, $p < .001$), supporting H3. These results offer empirical support for the dual-pathway model: students’ AM serves not only as a direct engine driving CES but also creates favorable conditions for deep engagement by enhancing their positive perception of school supportive initiatives.

**Table 4:** Main effect path coefficients

Hypothesis	Path	$\boldsymbol{\beta }$	S.E.	*t*	*p*	Result
H1	AM $\rightarrow$ CES	0.089	0.003	32.95	<.001	Supported
H2	AM $\rightarrow$ PSS	0.221	0.003	87.55	<.001	Supported
H3	PSS $\rightarrow$ CES	0.464	0.004	112.65	<.001	Supported

$\beta$ = standardized coefficient from design-corrected regression (svyglm) based on factor scores. S.E. = standard error

### Moderating effects of OECD membership and gender

The moderating effects of OECD membership and gender on all three core pathways are presented in Table 5. OECD membership significantly moderated both the AM $\rightarrow$ PSS path ($\beta = -0.048$, SE $= 0.005$, $t = -9.55$, $p < .001$) and the AM $\rightarrow$ CES path ($\beta = -0.057$, SE $= 0.007$, $t = -8.26$, $p < .001$), supporting H5b and H5a respectively. The negative coefficients indicate that the direct effects of AM on both PSS and CES are weaker in OECD countries compared to non-OECD countries. OECD membership did not significantly moderate the PSS $\rightarrow$ CES path ($\beta = 0.002$, SE $= 0.008$, $t = 0.30$, $p = 0.765$; H5c not supported).

**Table 5:** Moderating effects of OECD membership and gender

Moderator	Hypothesis	Path	$\boldsymbol{\beta }$	S.E.	*t*	*p*
OECD	H5a	AM $\times$ OECD $\rightarrow$ CES	$-0.057$	0.007	$-8.26$	<.001
OECD	H5b	AM $\times$ OECD $\rightarrow$ PSS	$-0.048$	0.005	$-9.55$	<.001
OECD	H5c	PSS $\times$ OECD $\rightarrow$ CES	0.002	0.008	0.30	0.765
Gender	H6a	AM $\times$ Gender $\rightarrow$ CES	$-0.026$	0.006	$-4.20$	<.001
Gender	H6b	AM $\times$ Gender $\rightarrow$ PSS	$-0.006$	0.005	$-1.17$	0.248
Gender	H6c	PSS $\times$ Gender $\rightarrow$ CES	0.011	0.007	1.61	0.113

$\beta$ = standardized coefficient from design-corrected regression (svyglm) based on factor scores. Reference groups: Non-OECD (for OECD) and Male (for Gender). Joint test of interaction terms: OECD: Working 2logLR $= 84.81$, $p < .001$; Gender: Working 2logLR $= 20.66$, $p < .001$. *S.E*. standard error

Gender significantly moderated the AM $\rightarrow$ CES path ($\beta = -0.026$, SE $= 0.006$, $t = -4.20$, $p < .001$), supporting H6a, with female students showing a weaker direct effect of motivation on CES. Gender did not significantly moderate the AM $\rightarrow$ PSS path ($\beta = -0.006$, SE $= 0.005$, $t = -1.17$, $p = 0.248$; H6b not supported) nor the PSS $\rightarrow$ CES path ($\beta = 0.011$, SE $= 0.007$, $t = 1.61$, $p = 0.113$; H6c not supported).

Notably, the PSS $\rightarrow$ CES pathway remained robust across all tested moderators—neither PR, OECD membership, nor gender significantly altered the strong positive effect of PSS on CES. This underscores the universal importance of supportive school environments in fostering deep learning.

### Moderating effects of PR

The moderating effects of PR on the three core pathways were tested using product terms between factor scores (see Table 6). PR significantly moderated the AM $\rightarrow$ PSS path ($\beta = 0.008$, SE $= 0.003$, $t = 2.80$, $p = 0.007$), supporting H4b. However, PR did not significantly moderate the AM $\rightarrow$ CES path ($\beta = 0.006$, SE $= 0.004$, $t = 1.46$, $p = 0.148$; H4a not supported) nor the PSS $\rightarrow$ CES path ($\beta = -0.002$, SE $= 0.006$, $t = -0.36$, $p = 0.717$; H4c not supported). These findings indicate that PR primarily functions as an amplifier in the initial stage of the process—enhancing motivated students’ ability to recognize and perceive PSS—but does not directly strengthen the translation of either motivation or perceived support into actual cognitive engagement strategies.

**Table 6:** Moderating effects of PR

Hypothesis	Path	$\beta$	S.E.	*t*	*p*
H4a	AM $\times$ PR $\rightarrow$ CES	0.006	0.004	1.46	0.148
H4b	AM $\times$ PR $\rightarrow$ PSS	0.008	0.003	2.80	0.007
H4c	PSS $\times$ PR $\rightarrow$ CES	$-0.002$	0.006	$-0.36$	0.717

$\beta$ = standardized coefficient from design-corrected regression (svyglm) based on factor scores. *S.E*. standard error

### Mediation analysis

The indirect effect of AM on CES through PSS was tested using structural equation modeling with bootstrap confidence intervals (see Table 7). The indirect effect was significant (estimate $= 0.103$, SE $= 0.001$, $z = 187.46$, $p < .001$, 95% bootstrap CI [0.102, 0.104]), accounting for 53.6% of the total effect (total effect $= 0.192$). The direct effect of AM on CES remained significant after accounting for the mediator (estimate $= 0.088$, SE $= 0.001$, $z = 79.61$, $p < .001$, 95% CI [0.086, 0.091]), indicating partial mediation.

**Table 7:** Mediation analysis with bootstrap confidence intervals

Effect	Estimate	S.E.	*z*	*p*	95% Bootstrap CI	Proportion
Indirect (AM $\rightarrow$ PSS $\rightarrow$ CES)	0.103	0.001	187.46	<.001	[0.102, 0.104]	53.6%
Direct (AM $\rightarrow$ CES)	0.088	0.001	79.61	<.001	[0.086, 0.091]	45.8%
Total	0.192	0.001	170.18	<.001	[0.189, 0.194]	100%

$N = 574{,}514$ after FIML. Bootstrap CIs based on 1,000 resamples (969 successful) using percentile method. S.E. and *z* are from the original MLR estimation. Percentages may not sum to 100% due to rounding

## Discussion

### Summary of findings

The findings collectively validate and elaborate the dual-pathway model presented in Fig. 1. Based on a large-scale multi-country sample spanning 80 educational systems, the analysis reveals a nuanced picture of learning transformation within the post-crisis “motivation–support–engagement” chain.

First, the confirmed sequential pathway (AM $\rightarrow$ PSS $\rightarrow$ CES) underscores the synergistic interplay between internal drive and external support. The study confirms that AM not only directly drives CES but also indirectly shapes more positive perceptions of PSS, accounting for over half of the total effect. This implies that motivated students are more inclined to interpret school support as valuable resources, and this positive perception, in turn, provides the psychological foundation for deep cognitive engagement strategies.

Second, the selective moderating role of PR refines our understanding of resilience in educational contexts. Contrary to the hypothesis that PR would uniformly amplify all pathways, PR only significantly moderated the AM $\rightarrow$ PSS path. This suggests that in the post-pandemic educational context, resilience primarily functions as a “perceptual amplifier”—helping motivated students recognize and interpret available school resources more favorably—rather than as a “utilization amplifier” that directly facilitates the conversion of resources into deep learning behaviors. One possible explanation is that the translation of perceived support into actual cognitive engagement strategies may depend more on pedagogical factors (e.g., instructional quality, task design) or contextual constraints (e.g., learning environment stability) than on individual psychological resources alone. This finding aligns with broaden-and-build theory [15], which posits that positive psychological resources broaden perception and build subsequent resources, but the actual deployment of those resources may require additional externally structured scaffolds.

Third, the significant moderating effects of OECD membership and gender on the AM $\rightarrow$ CES path introduce important nuances. The weaker direct effect of motivation on CES in OECD countries may reflect differences in educational structures or cultural values regarding intrinsic motivation, or potential ceiling effects in resource-rich environments. Similarly, the gender difference—with female students showing a weaker direct translation of motivation into engagement—may stem from variations in self-regulatory styles or differential responses to academic pressures. Critically, the non-significant moderation of the PSS $\rightarrow$ CES path by both OECD and gender underscores the universal importance of PSS in fostering deep learning, transcending national and gender boundaries. This aligns with self-determination theory’s assertion that the fulfillment of basic psychological needs is a universal prerequisite for optimal functioning [3].

### Theoretical implications

At the theoretical level, this study makes three primary contributions. First, it advances the conceptualization of educational resilience from a static “protective shield” [5] to a more dynamic “process moderator” that selectively facilitates specific stages of the learning process. By demonstrating that PR facilitates the perception of support but not the utilization of that support, the findings encourage a more granular approach to studying resilience mechanisms. Second, the study integrates Self-Determination Theory and Social Cognitive Theory to reveal a sequential motivational-cognitive process: AM shapes the subjective interpretation of the environment (PSS), which in turn fuels deep engagement (CES). Third, by testing and finding mixed support for the moderating role of macro-contextual variables, the study highlights the importance of distinguishing between individual psychological resources and contextual factors in shaping educational outcomes.

### Practical implications

The findings suggest that educational interventions could shift toward “resilience-sensitive” differentiated support. For students with lower resilience, interventions might focus on explicitly scaffolding the perception and interpretation of available support—making support more visible and interpretable. For students with higher resilience, interventions might focus on providing more autonomous and challenging learning environments that allow them to fully leverage their enhanced perceptual capacities. Furthermore, given the universal robustness of the PSS $\rightarrow$ CES pathway, investments in improving the quality and visibility of school support systems are likely to yield broad benefits across diverse student populations.

### Limitations and future directions

While achieving significant findings, this study has limitations that point to directions for future research. First, the cross-sectional design inherently limits causal inference. Although we constructed a sequential pathway and moderation model based on theory, the data cannot firmly establish causal directions. Future research should employ longitudinal designs to reveal dynamic interactions among motivation, perceived support, engagement, and resilience. Second, the reliance on self-report measures may introduce common method variance. Future studies could incorporate multi-modal data, such as teacher ratings or trace data from online learning platforms. Third, the AVE values for PSS, CES, and PR were below the conventional 0.50 threshold, reflecting the challenges of measuring complex psychological constructs with brief scales in large-scale international assessments. Fourth, the FIML approach assumes data are MAR; while this is more plausible than the missing completely at random assumption required for listwise deletion, we cannot rule out the possibility of non-ignorable missingness, particularly given the high missing rates on pandemic-specific items. Fifth, while we employed design-corrected regression for moderation tests, the two-step factor score approach may slightly attenuate interaction effect sizes compared to full latent interaction modeling.

Future research should also adopt a multisystem perspective to examine how resilience operates across individual, family, school, and community levels in post-crisis educational contexts [25]. Furthermore, it is important to distinguish the conceptualization of psychological resilience employed in this study from the related but distinct construct of academic buoyancy. Academic buoyancy refers to students’ capacity to successfully deal with everyday academic setbacks and challenges typical of ordinary school life [26]. The PR items used in PISA 2022 assess emotional control, adversity recovery, and stress coping in the context of the global COVID-19 pandemic—conditions that more closely align with resilience to significant adversity rather than everyday buoyancy. Future research would benefit from employing refined measures that can disentangle these two constructs within crisis learning contexts.

## Conclusion

By constructing and testing a dual-pathway model with PR as a selective moderator, this study conducted an in-depth multi-country empirical exploration of the intrinsic mechanisms of student learning engagement in the global post-crisis context. Based on a large-scale, design-corrected analysis of PISA 2022 data, the findings reveal that AM predicts CES both directly and indirectly through enhanced PSS. PR selectively amplifies the perception of support but does not directly facilitate the conversion of resources into engagement. The universal robustness of the support-to-engagement pathway across national development levels and genders underscores the foundational importance of supportive school environments.

As global education progresses into the “post-crisis era,” building truly resilient educational systems requires attention not only to the quantity of support resources but also to the psychological processes through which students perceive, interpret, and mobilize those resources. Investing in the cultivation of students’ PR—particularly their capacity to recognize and internalize available support—represents a strategic investment in educational equity and long-term efficacy.

## Data Availability

The datasets supporting the conclusions of this article are available in the OECD PISA 2022 repository, https://www.oecd.org/pisa/data/2022database/.
